# Natural variation of chronological aging in the *Saccharomyces cerevisiae* species reveals diet-dependent mechanisms of life span control

**DOI:** 10.1038/s41514-018-0022-6

**Published:** 2018-03-12

**Authors:** Paul P. Jung, Zhi Zhang, Nicole Paczia, Christian Jaeger, Tomasz Ignac, Patrick May, Carole L. Linster

**Affiliations:** Luxembourg Centre for Systems Biomedicine, University of Luxembourg, Esch-sur-Alzette, Luxembourg

## Abstract

Aging is a complex trait of broad scientific interest, especially because of its intrinsic link with common human diseases. Pioneering work on aging-related mechanisms has been made in *Saccharomyces cerevisiae*, mainly through the use of deletion collections isogenic to the S288c reference strain. In this study, using a recently published high-throughput approach, we quantified chronological life span (CLS) within a collection of 58 natural strains across seven different conditions. We observed a broad aging variability suggesting the implication of diverse genetic and environmental factors in chronological aging control. Two major Quantitative Trait Loci (QTLs) were identified within a biparental population obtained by crossing two natural isolates with contrasting aging behavior. Detection of these QTLs was dependent upon the nature and concentration of the carbon sources available for growth. In the first QTL, the *RIM15* gene was identified as major regulator of aging under low glucose condition, lending further support to the importance of nutrient-sensing pathways in longevity control under calorie restriction. In the second QTL, we could show that the *SER1* gene, encoding a conserved aminotransferase of the serine synthesis pathway not previously linked to aging, is causally associated with CLS regulation, especially under high glucose condition. These findings hint toward a new mechanism of life span control involving a trade-off between serine synthesis and aging, most likely through modulation of acetate and trehalose metabolism. More generally it shows that genetic linkage studies across natural strains represent a promising strategy to further unravel the molecular basis of aging.

## A metabolic block favoring long sweet life

A Sake yeast strain deficient in producing the protein building block serine lives longer than other yeast strains, especially when exposed to high glucose. A team led by Carole Linster at the University of Luxembourg found a broad variability of lifespan when analyzing more than fifty *Saccharomyces cerevisiae* strains isolated from around the world. Combining hundreds of lifespan measurements with genotype data from a progeny obtained by crossing the long-lived Sake strain and a short-lived collection strain, they identified two genes playing a pivotal role in causing the contrasting aging behavior of the parents: *RIM15*, when glucose was limiting and *SER1*, when glucose was plenty. *RIM15* is part of a signaling cascade also regulating aging in mammals; *SER1* revealed that a blockage in serine synthesis reprograms metabolism to favor glucose storage and long life.

## Introduction

Aging is a fundamental property of life. As it is a leading risk factor for many common diseases in humans, research on the molecular mechanisms involved in the aging process is expected to lead to a better understanding of age-associated diseases and to promote the development of health and/or life span extending strategies. In the past decades, fundamental mechanisms involved in the aging process have been extensively studied using different model organisms and more recent studies have shown that a number of pathways contributing to life span control are conserved across species.^1,2^ The unicellular eukaryotic organism *Saccharomyces cerevisiae* has transitioned over the past decades from being one of the most widely used model organisms to elucidate fundamental cellular processes through classical biochemical or genetic approaches to becoming a model of choice to pioneer emerging disciplines such as functional genomics and systems biology.^3,4^ More specifically in the aging field, research using both classical and more systematic approaches in *S. cerevisiae* has played a pivotal role in the discovery of major conserved longevity factors and pathways, such as the sirtuins and TOR signaling.^1^

Different approaches have been developed to systematically investigate chronological life span (CLS) in large yeast gene deletion collections, including outgrowth kinetics assays,^5,6^ barcoded competition-based assays,^7^ and fluorescence labeling.^8^ Surveys based on these approaches unraveled the implication of conserved signaling pathways, including the well-documented TOR/Sch9 and Ras/cAMP/PKA pathways,^1,9,10^ as well as of various genes associated with autophagy, chromatin modification, or mitochondrial function.^7,8,11^ To date, more than 1000 genes have been linked to CLS variation in the *Saccharomyces* Genome Database, underlining the importance of the genetic component in life span control and contributing to elucidate the basic cellular and molecular mechanisms that impact the aging process in a eukaryotic setting. However, limiting this research to the commonly used yeast deletion collections suffers from three major drawbacks. First, only loss-of-function mutations (gene deletions) are examined, which restricts the spectrum of phenotypic variation that can be observed. Second, recent studies showed that these collections often harbor secondary mutations in addition to the gene deletions, leading to heterogeneous populations in 56% of the strains.^12^ Ultimately, this can lead to potential misinterpretations of genotype–phenotype associations. And finally, all strains from deletion collections are isogenic to few laboratory strains, mostly deriving from the BY strain which has been shown to be phenotypically extreme for a number of traits and is certainly not representative of the natural diversity within the *S. cerevisiae* species.^13^

Natural isolates of *S. cerevisiae* present considerable genetic and phenotypic diversity^14,15^ and constitute therefore promising tools to uncover new longevity factors. Genome analyses of different *S. cerevisiae* strains revealed fluctuations in terms of gene and transposon content, gene copy number, as well as chromosome structure.^14–18^ Previously, two main segregating populations have been generated in *S. cerevisiae* from hybrids between the reference strain S288c (or the isogenic BY strain) and either a clinical strain isolated from an immunodepressed patient or a vineyard strain, described as S288c/YJM and BY/RM hybrids, respectively.^19,20^ These segregant collections have been used to investigate the genetic basis underlying various traits, including fitness variation in different conditions as well as variations in gene expression, translation or metabolic content.^19,21–23^ Concerning CLS, linkage mapping using the BY/RM cross identified a single-nucleotide polymorphism in the *BUL2* gene affecting telomere maintenance as modulating chronological aging.^24^ However, both crosses involve the reference laboratory strain, which harbors multiple auxotrophic markers and in addition has been shown to contain highly pleiotropic *HAP1* and *MKT1* alleles that cause important confounding effects in Quantitative Trait Loci (QTLs) mapping.^21,25^

To circumvent the biases associated with the S288c reference strain, we investigated CLS variation within a population of 58 natural isolates of *S. cerevisiae*.^17^ In addition, various dietary carbon sources were used to determine how different environmental conditions affect aging mechanisms in different genetic backgrounds. CLS profiling in this collection revealed a broad variability of aging behaviors across the strains and across the conditions tested, hinting at a multitude of genetic and environmental factors at play to control chronological aging. We further dissected the genetic basis of CLS, and its dependence on the environment, through linkage mapping in a large progeny generated by crossing two isolates with contrasting chronological aging behaviors. Using two distinct mapping strategies, we identified two major QTLs through our segregating population. Strikingly, the detection of each of these aging QTLs depended largely on the initial dietary conditions used to launch the cultivations. Based on these findings, we propose a model of diet-dependent differential control of CLS, where the nutrient-sensing pathway is predominantly involved under glucose restricted conditions, whereas another mechanism involving a metabolic state characterized by lower acetate accumulation and increased trehalose synthesis in response to serine auxotrophy seems to play a major role in CLS control under glucose rich conditions.

## Results

### CLS variation within the *S. cerevisiae* yeast species

**Fig. 1:**
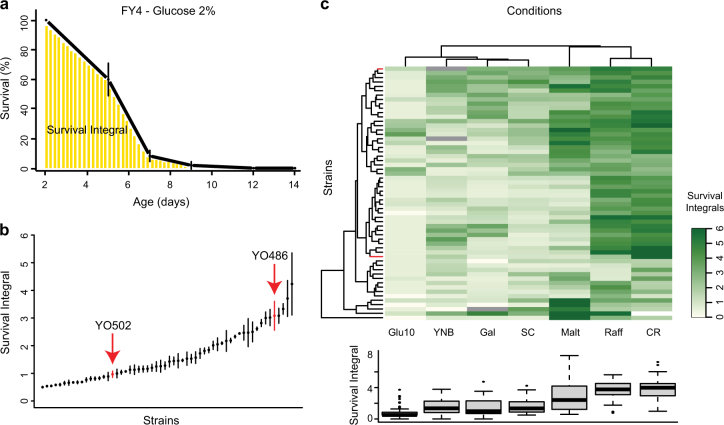
Chronological life span variation in a natural yeast strain collection. **a** Representative chronological life span (CLS) assay, based on determination of the Survival Integral (SI), for the FY4 strain in our standard SC medium. **b** CLS determination for our natural strain collection in SC condition. Arrows indicate the parental strains of the “sake × tecc cross”. **c** Heatmap and boxplot representation of CLS variation within the natural strain collection in seven different conditions. The color gradient represents SI variation with dark and light green corresponding to long and short life span, respectively. Gray rectangles in the heatmap indicate no growth of the corresponding strains in the respective conditions. Strains depicted in red correspond to the parental strains of the “sake × tecc cross”

In addition to five lab strains (BY4741, FY4, W303a, FL100, and the diploid FY_2n), 53 natural *S. cerevisiae* isolates (Table S1) were screened for CLS variability in seven different conditions. The latter corresponded to synthetic complete medium supplemented with different glucose concentrations (0.5% (also known as Calorie restriction or CR), 2% (SC), or 10% (Glu10)) or alternative carbon sources at 2% (galactose (Gal), maltose (Malt), or raffinose (Raff)). Moreover, the impact of amino acid supplementation (as well as adenine, uracil, myo-inositol, and aminobenzoic acid) was investigated by comparing SC and YNB (minimal medium containing only ammonium sulfate and 2% glucose) media. As described previously,^6^ CLS was quantified by calculating the Survival Integrals (SIs) based on a high-throughput outgrowth kinetics assay (Fig. 1a). In the standard SC condition, natural variants showed a broad CLS variability, with SIs ranging from 0.50 to 4.23 (Fig. 1b). Hierarchical clustering according to the seven aging conditions identified two main groups of environments leading to shortened or prolonged life span based on the median life span across all strains tested (Fig. 1c). On average, a 3-fold life span extension was observed under CR compared to the SC condition (Fig. 1c; *p*-value < 2.2 × 10^−16^). The shortest CLS was observed on average when cultivations were launched in the presence of 10% glucose (Fig. 1c).

Interestingly, the presence of raffinose instead of 2% glucose led to a life span extension comparable to the one observed under CR (Fig. 1c; *p*-value > 0.05, Table S2). Linear correlation analysis between these two conditions revealed a good positive correlation (Pearson coefficient *R* = 0.52), possibly suggesting similar genetic mechanisms of life span control while aging in the presence of low glucose or raffinose. Aging in SC condition was correlated with the Glu10 and Gal conditions (*R* = 0.43 and 0.48, respectively) across the strain collection. The average CLS behavior of our strain collection did not differ significantly between the SC and YNB media (*p* = 0.94; Fig. 1c) and no significant correlation was observed between these conditions (Table S3). This is in contradiction with previous studies mostly using reference laboratory strains which indicated that amino acid content in the medium modulates aging in *S. cerevisiae*.^26^ All pairwise condition–condition correlations and statistical analyses are summarized in Supplementary Tables S2 and S3. Taken together, these data indicate that chronological aging is a complex trait that is highly influenced by nutritional conditions.

### CLS variation within the “sake × tecc cross”

**Fig. 2:**
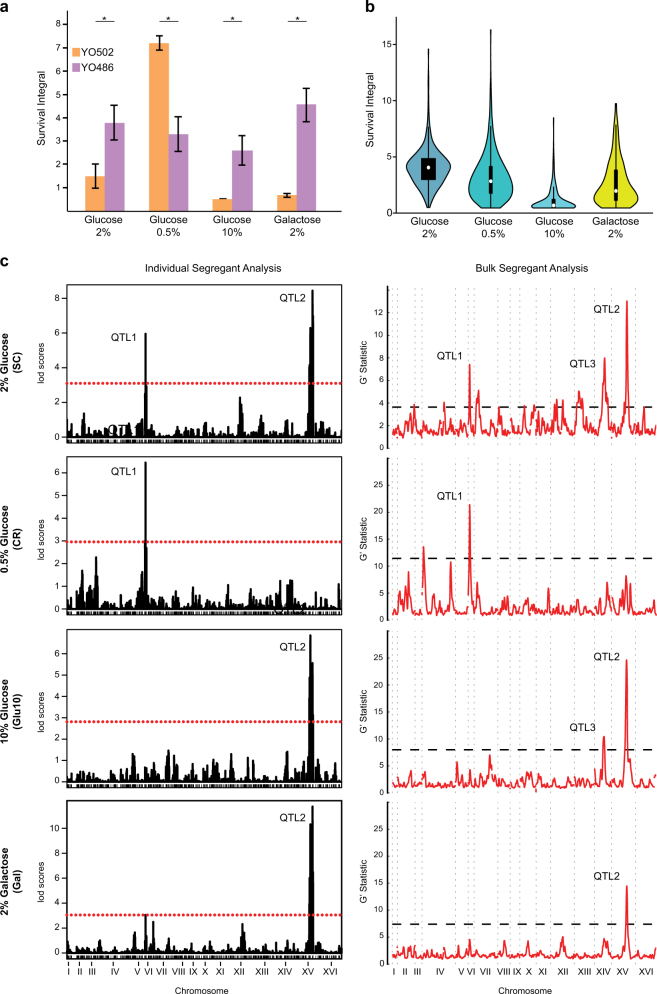
CLS variation across the “sake × tecc cross” reveals different aging QTLs depending on growth conditions. **a** Comparison of SIs between the parental strains of the “sake × tecc cross” shows significant differences; Asterisk, significantly different by Student’s *t*-test, *p*-value < 0.05. Values are mean ± SDs. **b** Violin plot representation of survival integrals determined for the “sake × tecc cross” segregants in the indicated conditions. **c** QTL mapping using Individual Segregant Analysis (ISA, left column) and Bulk Segregant Analysis (BSA, right column) strategies for CLS data obtained when strains were aged in SC medium containing 2% glucose (SC), 0.5% glucose (CR), 10% glucose (Glu10), and 2% galactose (Gal). For ISA, the dotted red line represents significance thresholds corresponding to *p*-values of 0.05. The black dashed line in the BSA panels, indicating the G′ statistic significance threshold, corresponds to a false discovery rate of 0.05

To identify specific genes responsible for CLS variation in at least part of our strain collection, we used a segregating population generated by Aimée Dudley’s laboratory (Pacific Northwest Research Institute, Seattle, USA), comprising 488 haploid spores derived from two natural strains (i.e., YO486 and YO502), hereafter referred to as “sake × tecc cross”.^27^ These two strains were selected based on contrasting aging phenotypes, with YO486 being long-lived in SC, Glu10, and Gal conditions, but short-lived in the CR condition as compared to the YO502 strain (Fig. 2a). In contrast, growth trait profiling of our natural strain collection across 26 conditions on solid media (Table S4) showed a reasonably similar phenotypic behavior of the two parental strains (Fig. S1).

For each haploid offspring of the “sake × tecc cross”, we determined CLS and growth parameters (specific growth rate and yield of biomass) in the SC, CR, Glu10, and Gal conditions (Fig. 2b and Fig. S2). Taking into account the data obtained across all environments tested, weak correlation was found between CLS and specific growth rate (*R* = −0.19), but a relatively strong negative correlation (*R* = −0.56) was calculated between the CLS and the yield of biomass calculated for the entire progeny (Fig. S2a). However, similar analyses performed for each condition individually revealed a negative correlation between both the growth rate and the yield of biomass and CLS for all conditions, except under caloric restriction (Fig. S3). In addition, pairwise comparisons of the SIs for the four tested conditions showed that the CR condition correlates only weakly with the other conditions (Pearson correlation coefficients between −0.08 and 0.19), whereas positive correlations were found between the SC and Glu10 conditions as well as the Gal and Glu10 conditions (*R* = 0.52 and 0.29, respectively) (Fig. S2b). Together these observations indicate potentially shared CLS control mechanisms under rich dietary conditions, whereas distinct pathways are at play when dietary resources are restricted from the start of the cultivation. For each condition, distribution of the SIs revealed a Gaussian-like curve, although normality was rejected (Shapiro–Wilk test, *p* < 1.10^−14^) likely due to the inability to measure SI values lower than 0.5 for short-living strains using our CLS assay. Transgressive segregation was detected for each condition, as part of the offspring displayed extreme short-living or long-living phenotypes, surpassing those observed for the parental strains (Fig. S4).

To map genetic loci associated with CLS variation, we used two different approaches: Individual Segregant Analysis (ISA) and Bulk Segregant Analysis (BSA). ISA is a straightforward, but rather laborious method to map QTLs as it is based on the use of segregating populations in which each single haploid strain needs to be genotyped and phenotyped. Here, QTL mapping based on ISA was performed using the R/qtl package, genotyping data previously obtained for all the “sake × tecc cross” segregants by RAD sequencing^27^ and the SI values generated during this study for all the segregants using our high-throughput CLS assay.^6^ BSA relies on allele frequency analysis in pools of segregant strains characterized by extreme phenotypes. Here, two pools or “bulks” were analyzed for each investigated condition, each comprising the 50 segregants with the highest or shortest CLS (Fig. S4). In the 2% glucose condition, both the ISA and BSA strategies mapped two major QTLs: one located on chromosome VI (QTL1) and another one on chromosome XV (QTL2). BSA allowed the identification of an additional strong QTL on chromosome XIV (QTL3) (Fig. 2c). Interestingly, the detection of those QTLs heavily depended on the environmental conditions: while QTL1 and QTL2 were both mapped in the standard condition, only QTL1 was detected under CR and only QTL2 was detected in the Glu10 and Gal conditions; QTL3 was only detected (using BSA) in standard and Glu10 conditions (Fig. 2c). The observation that QTL1 was detected, at the same sugar concentration (2%), in the presence of glucose (SC condition) but not galactose, suggested a glucose repression-dependent mechanism;^28^ the absence of QTL1 in the presence of 10% glucose, however, questions this interpretation.

**Fig. 3:**
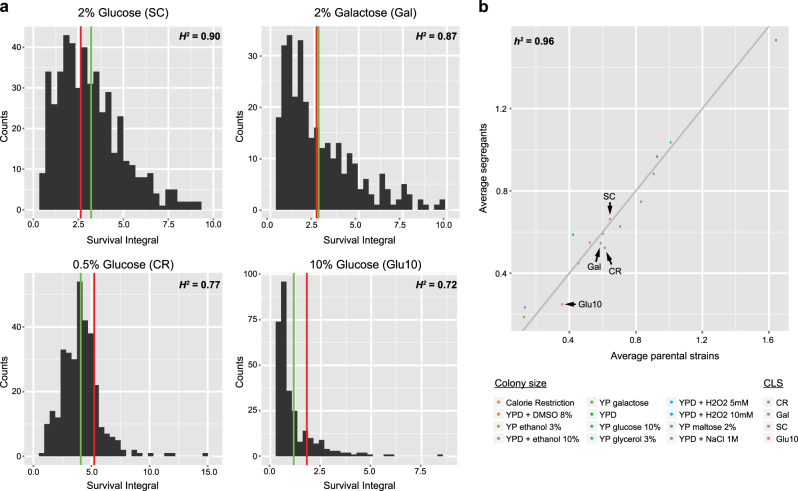
Heritability analyses of CLS in a segregating yeast population. **a** Broad-sense heritability *H*^2^ was estimated as described in Supplementary Information. Distributions of SIs determined for the entire “sake × tecc cross” are shown for the four tested growth conditions. Average of SIs for parental strains and the segregating population are depicted in red and green, respectively. **b** Narrow-sense heritability *h*^2^ was estimated by comparing the averages of the CLS data obtained for the parental strains and the segregants in the indicated conditions. Average colony sizes obtained for parental and segregant strains in 12 growth conditions were also compared. For a better chart clarity, CLS data are represented at a 1/5 ratio for SC, Glu10, and Gal conditions and a 1/10 ratio for the CR condition. The *h*^2^ was estimated based on the parent-offspring regression taking into account all the data points

To further deepen our understanding of the genetic architecture underlying CLS control, we also analyzed the heritability of the CLS trait in the “sake × tecc cross”. The broad-sense heritability (*H*^2^) is defined as the ratio of total genetic variance (*V*_G_), resulting from the sum of additive genetic effects (*V*_A_), dominance effects (*V*_D_), gene–gene interactions (*V*_I_), and gene–environment interactions (*V*_E_), to total phenotypic variance (*V*_P_) (*H*^2^ = *V*_G_/*V*_P_, where *V*_G_ = *V*_A_ + *V*_D_ + *V*_I_ + *V*_E_).^29,30^ In our study, there was no dominance effect given that only haploid segregants were used for linkage mapping. Moreover, as each condition was analyzed individually, gene–environment interactions could also be neglected. Thus, *H*^2^ only reflects the effects of additive genetic factors and of epistasis on CLS (*V*_G_ = *V*_A_ + *V*_I_). As shown in Fig. 3a, a high *H*^2^ value (0.72–0.90) was observed in all four conditions studied. Next, we estimated the narrow-sense heritability (*h*^2^), representing only the additive genetic effects on phenotypic variation (*h*^2^ = *V*_A_/*V*_P_), by calculating the parent-offspring regression for the CLS phenotype^29^ as well as for colony size determined in 12 conditions on solid media (Table S5). Through deep investigation of heritability features within the BY/RM segregating population phenotyped in various conditions (CLS data obtained from this cross^24^ were not included), it has recently been reported that trait variation is mainly due to additive genetic factors and not epistatic interactions.^30,31^ Here, the *h*^2^ for CLS and growth traits was calculated to be ~0.95 and ~0.97, respectively; *h*^2^ corresponded to ~0.96 when all phenotypic data were pooled together (Fig. 3b). This indicated that the CLS phenotype, at least in the four tested conditions, is mainly controlled by additive genetic effects.^29^ Combined, these two heritability analyses suggested that, for a given environment, chronological aging is strongly controlled by genetic variation, but with little or no contribution by epistatic effects.

### *RIM15* is a major effector of CLS variation in restricted dietary conditions

Our BSA approach allowed us to drastically narrow the size of the QTL regions when compared to the ISA method. BSA analysis showed that QTL1 contained five genes over a ~13 kb region on the left arm of chromosome VI (compared to 28 genes over a ~55 kb region after ISA), and two candidate genes, *RIM15* and *MIL1*, were identified. Indeed, sequence analyses revealed pre-mature stop codons in those genes (W172X and D258X for *RIM15* and *MIL1*, respectively) that likely abolish corresponding protein functions in the long-lived YO486 strain. *RIM15* encodes a protein kinase acting as regulator of various transcription factors including notably the stress responsive transcriptional activators Msn2 and Msn4, in both the TOR/Sch9 and Ras/cAMP/PKA pathways, known as key players of aging regulation via nutrient-sensing.^9,10,32^ *MIL1* encodes a putative lipase involved in the regulation of clathrin adapter complex recruitment to the Golgi/early endosome membrane.^33^

**Fig. 4:**
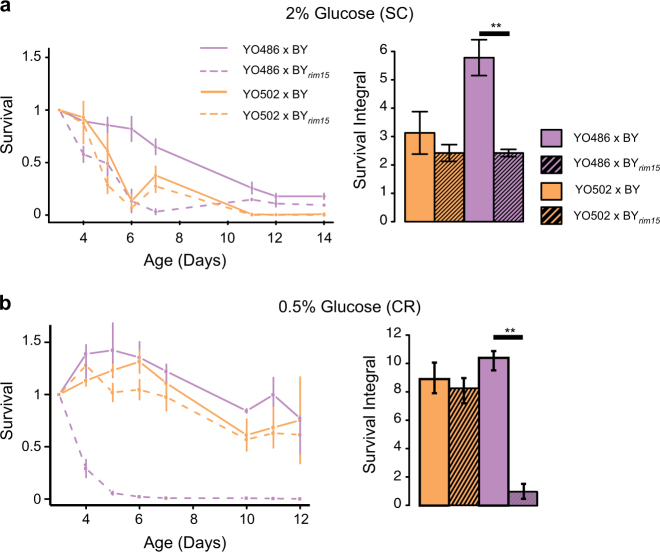
Validation of *RIM15* as an aging gene by a non-complementation approach. Survival curves and survival integrals were determined using an outgrowth kinetics assay for the indicated hybrid strains in SC medium containing 2% glucose (**a**) and 0.5% glucose (calorie restriction) (**b**). The results correspond to mean ± SDs for three biological replicates. Double asterisk, significantly different from control strain by Student’s *t*-test, *p*-value < 0.005

We used a non-complementation test^34^ to validate candidate genes by crossing both parental isolates with strains isogenic to the BY background, either with or without deletion of the gene of interest. *RIM15* has been reported to decrease the CLS in calorie-restricted condition when knocked out.^35^ Hence, a functional *RIM15* allele from a parental strain should complement the loss-of-function of the deleted *RIM15* allele in the hybrids. By contrast, a decrease in CLS would be expected if the mutated *RIM15*_YO486_ allele were to play a causal role in aging variation. In both SC and CR conditions, the BY_rim15_/YO486 hybrid showed a decreased CLS compared to the BY/YO486 diploid (Fig. 4), meaning that the *RIM15*_YO486_ allele did not complement the deleted BY allele. By contrast, the *RIM15*_YO502_ allele complemented the deleted BY allele in the diploid BY_rim15_/YO502 (Fig. 4). No statistically significant differences were detected when the same non-complementation tests were performed for the *MIL1* gene (Fig. S5).

These results suggest that the *RIM1*5 allele of the YO486 parental strain prevents the life span extension normally observed under CR (Fig. 2a). It can be noted that while the CLS of the haploid YO486 strain is indeed not extended in CR condition as compared to the standard SC condition, the hybrid BY/YO486 strain is following the more generally observed trend of life span extension under CR (Fig. 4). This difference is probably mostly due to the presence of the functional BY allele of *RIM15* in the hybrid BY/YO486 diploid background, as the CR-dependent CLS extension is abolished in the BY_rim15_/YO486 hybrid (Fig. 4). The fact that under rich dietary conditions, the haploid YO486 strain is long-lived as compared to the YO502 strain (Fig. 2a) indicated, however, that a *RIM15*-independent mechanism, likely involving the QTL2 locus, seemed to have a dominating effect on CLS determination under these conditions.^35^

### *SER1* is involved in CLS regulation under high glucose and galactose conditions

**Fig. 5:**
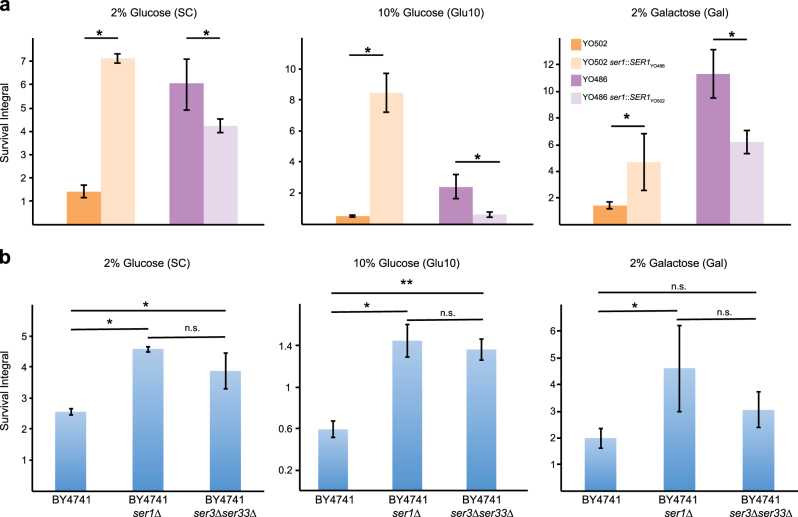
Validation of *SER1* as an aging gene. **a** Survival integrals for allele-swapped strains were determined using an outgrowth kinetics assay for the indicated strains in SC medium containing 2% glucose, 2% galactose, and 10% glucose. **b** Survival integrals for *ser1*Δ and *ser3*Δ*ser33*Δ deletion mutants and the isogenic BY4741 strain. Results correspond to mean ± SDs for three biological replicates. n.s. not significantly different; *p < 0.05; **p < 0.005 according to Student’s *t*-test

QTL2 was mapped on the right arm of chromosome XV at ~700 kb from the centromere, where its size was calculated to be ~10, 14, and 23 kb in Glu10, SC, and Gal conditions, respectively, using the BSA approach. In these conditions, the CLS profiles of the “sake × tecc cross” parental strains indicated that the YO486 allele of the causal gene in QTL2 should lead to a prolonged CLS as compared to the YO502 allele. Non-complementation tests performed for eight candidate genes in QTL2 containing non-synonymous mutations (*BFR1*, *FYV12*, *GAC1*, *MGM1*, *NPT1*, *SLK19*, *THI72*, *ULS1*) failed to demonstrate a causal link between those genes and CLS. The *SER1*_YO486_ allele, located at ~2, 20, and 12 kb from the QTL2 peak in Glu10, SC, and Gal, respectively, has been found to affect a number of phenotypes in this sake strain.^27^ Using allele replacement, we validated *SER1* as a QTL2-associated causative gene for the chronological aging phenotype in these three conditions: the *SER1*_YO486_ allele prolonged the CLS in the YO502 strain, whereas the *SER1*_YO502_ allele shortened CLS in the YO486 background (Fig. 5a). In contrast, no significant differences were found in CR medium (data not shown). These results suggest that *SER1* may be the only gene contributing to CLS variation in QTL2.

Although the metabolic function of *SER1* is established (aminotransferase catalyzing the formation of 3-phosphoserine from 3-phosphohydroxypyruvate in the serine biosynthesis pathway starting from the glycolytic intermediate 3-phosphoglycerate),^36^ a role for this gene in chronological aging has never been reported before. Sequence analysis revealed one non-synonymous mutation (G78R) in the *SER1*_YO486_ allele. This glycyl residue is highly conserved across species; according to the NCBI conserved domain database,^37^ Gly78 is located in the homodimer interface and followed directly by another conserved glycyl residue that is involved in pyridoxal 5′-phosphate binding, essential to the enzymatic activity of *SER1*. Growth comparison of the allele-swapped strains in YNB supplemented or not with serine confirmed that this mutation confers auxotrophy towards serine (Fig. S6a), as previously described.^27^ In addition, we also estimated growth rates and yields of biomass for these strains in SC, CR, Glu10, and Gal conditions (Fig. S6b). Strikingly, the *SER1*_YO486_ allele decreases growth rate and biomass in the YO502 strain in all tested conditions, whereas the *SER1*_YO502_ allele increases these features in the YO486 strain. These opposing effects of the *SER1*_YO486_ allele on fitness and CLS (negative effect on fitness, positive effect on CLS, at least non-calorie-restricted conditions) is reminiscent of the negative correlation observed in non-CR conditions for the entire segregating population between growth and CLS (Fig. S2 and Fig. S3).

Since *SER1* had, to the best of our knowledge, never been linked with CLS before, it was important to determine whether this newly observed association is background-specific and/or allele-specific. Deletion of *SER1* in the BY4741 background led to a significant increase in CLS in non-CR conditions (Fig. 5b). A similar trend was observed for a *ser3*Δ /*ser33*Δ mutant, deficient in the first step of the serine synthesis pathway. These results indicated that the life span extension caused by the *SER1*_YO486_ allele involves a more general, non-background-specific loss-of-function mechanism in which deficient serine synthesis promotes long life.

### *SER1* deficiency prevents acetate accumulation and promotes trehalose synthesis during aging

**Fig. 6:**
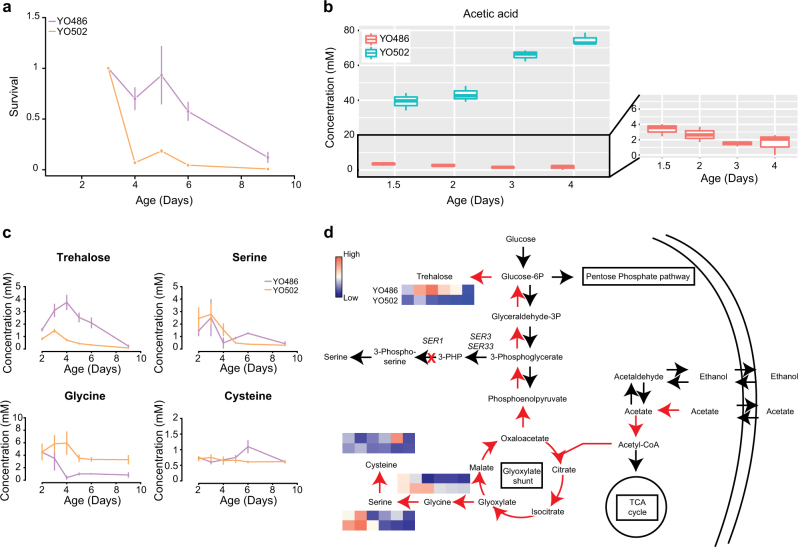
Metabolome variation during aging for the parental strains of the “sake × tecc cross”. **a** CLS profiles obtained for YO486 (violet) and YO502 (orange) from flask cultivations in SC medium supplemented with 2% glucose. The aging cultivations were sampled at different time points for endo-metabolome and exo-metabolome analyses. **b** Acetic acid was quantified by GC-MS in the extracellular medium of the YO486 (in red) and YO502 (in blue) strains. **c** Intracellular trehalose, serine, glycine, and cysteine concentrations were also determined during aging in the two strains using LC-MS. **d** Schematic overview showing the metabolites quantified in this study within the known metabolic network of *S. cerevisiae*. The endo-metabolome data is represented by heatmaps where red and blue squares correspond to high and low amounts of each metabolite, respectively, at the different time points. The main serine synthesis pathway, starting from 3-phosphoglycerate, is also shown. The first and second reactions in this pathway are catalyzed by the Ser3 (or its paralog Ser33) and Ser1 enzymes, respectively. It is the latter enzyme that is deficient in the YO486 strain (as indicated by the red cross). 3-PHP, 3-phosphohydroxypyruvate

Extracellular acetic acid accumulation and medium acidification have been associated with CLS decrease in yeast,^38^ although the role of acetic acid remains controversial.^39^ We determined the pH for both the YO486 and YO502 strains after 3 days of aging in the four conditions of interest. Acidification was measured for both strains and to a similar extent in all conditions but the CR condition, where the pH had not changed or had even slightly increased (Table S6), as described previously for different lab strains.^38^ In stark contrast, time-series analyses of acetate in SC medium during aging showed major differences between the YO486 and YO502 strains, the latter accumulating increasing concentrations of this organic acid while the former started out with more than 10-fold lower amounts, which then decreased over time (Fig. 6). After 3 days of aging, the YO502 strain had accumulated 40-fold more acetate in the medium than the YO486 strain.

Switching the medium (SC with 2% glucose) at day 3 to water increased lifespan in the YO502 strain, but decreased it in the YO486 strain (Fig. S7). This indicated that extracellular acetate (or other extracellular factors) may contribute to the different CLS behavior of both strains in this condition.

To determine whether the observed differences in extracellular acetate accumulation are specifically linked to the *SER1* gene, we integrated the *ser1* loss-of-function allele of the YO486 strain into the prototrophic FY4 strain (to avoid any bias due to auxotrophies in the BY strains) at the *SER1* locus. The resulting strain, designated FY4ser1_YO486_, is auxotrophic towards serine. Comparison of the medium pH during aging between the FY4 and FY4ser1_YO486_ strains showed an acidification in all conditions even if to a lesser extent in the CR condition (Table S6). This acidification was comparable in both strains. In contrast, acetate accumulation was ~30-fold higher in the FY4 strain than in the FY4ser1_YO486_ strain, corroborating our observations for the YO486 and YO502 strains and suggesting a specific association between *SER1* loss-of-function and low acetate accumulation during aging (Fig. S8).

Lower extracellular acetate accumulation has been linked to higher intracellular trehalose levels in a long-lived *sch9* mutant.^40^ Accordingly, we found in this study that the low acetate accumulating strains YO486 and FY4ser1_YO486_ maintain higher intracellular levels of trehalose during aging (Fig. 6c and Fig. S8c). The fact that the FY4 ser1_YO486_ strain mimicked the YO486 strain in terms of acetate and trehalose levels, allows to clearly dissociate this metabolic behavior from the *RIM15* loss-of-function that is present in the YO486 strain but not in the FY4 strain. It is interesting to note that both strains had the capacity to increase their trehalose storage during the first 3–4 days of aging, after which the levels of this dissacharide decreased, just before the onset of decreased survival (Fig. 6 and Fig. S8). Time profiles of intracellular serine and cysteine levels during aging showed overlapping curves for the YO486 and YO502 (or FY4 and FY4ser1_YO486_) strains until day 4. However, the YO486 and FY4ser1_YO486_ strains were able to increase their serine and cysteine levels thereafter, reaching a peak at day 6 when trehalose was being consumed. This apparently paradoxical finding can readily be explained by the existence, in addition to the main serine synthesis pathway that is blocked in these strains, of an alternative (glucose-repressed) pathway that involves conversion of glyoxylate to glycine, a serine precursor.^41^ These observations indicate that a genetic blockage in the main serine synthesis pathway from the beginning of life predisposes yeast cells to survive longer, apparently because of a metabolic reprogramming that prevents extracellular acetate accumulation and favors the build up of high intracellular trehalose stores, potentially involving increased activity of the glyoxylate shunt.

## Discussion

Using our recently developed high-throughput approach to quantify CLS^6^ in a collection of 58 strains across 7 different conditions, we observed a broad aging variability suggesting the implication of diverse genetic and environmental factors in CLS control. Our work differs from the bulk of aging research done in yeast so far by mainly using prototrophic non-laboratory strains, thereby avoiding biases caused by auxotrophies and pleiotropic alleles in S288C-derived strains and potentially increasing the discovery potential for new aging genes. A previous QTL analysis of CLS performed within the BY/RM cross, where derived haploid strains were aged in YPD medium, showed that one of the known pleiotropic genes in the S288C strain, namely *BUL2*, is involved in life span regulation.^24^ A loss-of-function mutation (F883L point mutation) in the reference BY strain decreases CLS through an increase of amino acid permease activity and amino acid uptake.^24^ To identify genes causally involved in CLS modulation independent of *BUL2*, we used two different linkage mapping strategies using a segregating population derived from a cross (sake × white tecc^27^) of two natural strains (YO486 and YO502; neither of those strains carries the F883L loss-of-function *BUL2* allele). Both mapping approaches identified two major aging QTLs depending on the type and/or concentration of carbohydrate source present in the medium. The results obtained suggested that QTL1 mainly modulates aging in calorie-restricted condition, whereas a QTL2-dependent mechanism becomes predominant as carbohydrate concentrations increase.

QTL1, located on chromosome VI, was detected in the presence of 0.5 and 2% glucose, but not in 10% glucose or 2% galactose. Gene validation within this QTL confirmed that *RIM15*, encoding a protein kinase on which both the TOR/Sch9 and Ras/cAMP/PKA pathways converge,^9,10,32^ is a gene causally affecting CLS in the presence of low to moderate glucose concentrations. *RIM15* has indeed been shown to support normal chronological aging by inducing several aspects of entry into quiescence upon nutrient limitation.^42,43^ Here, we rediscover this role of *RIM15* using a completely different approach, providing thereby a proof of principle that our linkage mapping strategies can be used to further elucidate molecular mechanisms of chronological aging. The importance of testing several environments is nicely illustrated, as *RIM15* was only identified as an aging gene in two of the conditions tested. Specifically, we showed here that a nonsense mutation in the *RIM15* allele of the YO486 strain (W172X) leads to shortened CLS under CR. However, our data also suggested that a second major genetic factor controls CLS in this strain and that the effect of the latter becomes increasingly important with increasing glucose concentration or in the presence of an alternative carbon source like galactose.

Testing gene candidates associated to the second major aging QTL, this factor was pinned down to the *SER1* gene, which had not been associated so far with aging in previous large-scale surveys of yeast deletion collections.^5,7,8^ We could show, however, that this link exists beyond the specific natural isolates studied here and involves a more general positive connection between serine auxotrophy and life span under non-CR conditions. Similarly, it has been shown by others that *leu2Δ0* and *met15Δ0* mutations in the BY4741 strain, leading to auxotrophies toward leucine and methionine, modulate life span.^44–46^ Amino acid homeostasis in general may thus play a key role in life span control. Indeed, high amounts of isoleucine, valine, threonine, leucine, and glutamic acid have been found to increase CLS in the typical BY lab strains, while in the case of methionine, CLS extension is triggered by restriction of this amino acid.^44–46^ These effects seem to be mediated by different mechanisms depending on the amino acid, involving processes such as autophagy, vacuolar acidification, or oxidative stress resistance.

Intriguingly, none of these studies highlighted serine as an aging modulator so far. One hypothesis put forward to explain life span dependency on leucine is based on codon abundance.^44^ Indeed, leucine codons show the highest relative abundance in the annotated protein-coding genes of the yeast genome and a limiting amount of leucine might inhibit the synthesis of proteins required for the stationary phase survival.^44^ Based on our analysis, serine is the second most frequent amino acid in yeast proteins encoded by the nuclear genome (Fig. S9). Serine is also a precursor for phospholipid biosynthesis and it has recently been demonstrated that the mitochondrial lipidome is involved in life span modulation.^47^ Somewhat surprisingly, we found in the present study that serine auxotrophy actually favors stationary phase survival. It is important, however, to remind that more than 40 years ago, Ulane and Ogur coined the term of “conditional auxotrophy” in the context of serine biosynthesis in yeast.^41^ Indeed, in addition to the main serine synthesis pathway starting from the glycolytic intermediate 3-phosphoglycerate and involving in the second step the Ser1 protein for transamination of 3-phosphohydroxypyruvate into 3-phosphoserine, a secondary pathway allows for serine synthesis via transamination of glyoxylate to glycine.^41^ The latter pathway is glucose-repressed, but allowed to explain in those early studies why strains that were auxotrophic for serine in glucose media, became independent on serine supplementation during growth on acetate. In our study, we showed that the loss-of-function *SER1* allele carried by the YO486 strain and leading to a blockage of the primary 3-phosphoglycerate-dependent serine synthesis pathway, confers prolonged CLS under non-CR conditions. Given the low extracellular acetate levels and the increased intracellular trehalose levels also measured in strains carrying the *SER1*_YO486_ allele, we speculate that the corresponding metabolic blockage leads to a metabolic reprogramming that favors acetate consumption and trehalose formation, possibly through increased activities in the glyoxylate shunt (and gluconeogenesis) needed to maintain a certain pool of serine in the cell (Fig. 6d, S8d).^41,48^ The resulting decreased acetate accumulation and increased trehalose stores likely contribute or may even entirely explain the life span extension observed in *SER1*-deficient strains. While acetate and trehalose have already been linked to CLS in numerous studies,^40,49^ this is the first time that those metabolites are proposed to participate in a mechanism for CLS extension in connection with serine synthesis.

Heritability analysis of chronological aging in the “sake × tecc cross” indicated that this phenotype results mainly from additive effects of individual genes and that epistasis plays only a minor role; this was recently corroborated by similar observations for 20 other quantitative traits in *S. cerevisiae*.^31^ Thus, theoretically, we should be able to predict aging solely from genome sequences. In practice this remains limited by the number of aging genes known so far, our understanding of the functional impact of single gene mutations, and the extent of our knowledge on the interplay between genetic and environmental factors in CLS regulation. In this study, we have identified two main genetic factors regulating CLS in a sugar content-dependant manner in two natural isolates. Follow-up investigation on the minor QTLs also found here could lead to the identification of additional aging genes. Notably, QTL3 on chromosome XIV seems of great interest as it has been highlighted in both the SC and Glu10 conditions.

Finally, in order to develop translational strategies that improve human health by delaying the onset or the progression of age-related diseases, significant research efforts are directed toward trying to mimic CR by nutritional supplementation of small molecules (e.g., rapamycin) that target conserved pathways (e.g., TOR pathway).^50^ Our study indicates that the life span extension caused by decreased TOR signaling can be outclassed by other mechanisms when the diet contains high amounts of sugar or when alternative sugar types are consumed. Unbiased genetic mapping approaches in various model organisms carried out under dietary conditions that more closely resemble human nutrition may further push the boundaries for the discovery of small molecules slowing down the aging process.

## Methods

### Strains and media

The list of strains used in this study is given in Table S1. A progeny of 488 haploid strains has been obtained through the cross of two parental strains isolated from Sake fermentation in Japan (YO486, deriving from UC5^51^) and from a White tecc tree in Ethiopia (YO502, also known as DBVPG1853^52^).^27^

CLS and growth analyses were carried out in minimal YNB (Yeast Nitrogen Base with ammonium sulfate from MP Biomedicals, 6.7 g/L) medium or synthetic complete medium (YNB containing SC amino acid mixture from MP Biomedicals at 2 g/L; Table S7) supplemented with 0.5% glucose (CR), 2% glucose (SC), 10% glucose (Glu10), 2% galactose (Gal), 2% raffinose (Raff), or 2% maltose (Malt). For growth phenotyping on plates, colony size was measured on solid YPD (10 g/L Yeast Extract, 20 g/L Bactopeptone, 20 g/L Glucose, 20 g/L Agar) supplemented with various compounds; as indicated, conditions where glucose was supplemented at concentrations differing from 2% or replaced by alternative carbon sources were also tested (Tables S4 and S5).

### Growth assays

Growth characteristics of strain collections were determined based on liquid microcultures performed in 384-well plates using the GATHODE software.^6^ At least three biological replicates were used for each measured growth parameter. Trait profile analysis of the natural isolate collection was performed on solid media. Using a 384 matrix, cells were transferred from a YPD master plate to 26 different conditions (Table S4) using a pinning robot (RoToR, Singer instruments). After 48 h incubation at 30 °C, plates were scanned with the EPSON V700 Photo scanner at a resolution of 300 dpi. Size and circularity of the colonies were calculated for six biological replicates using the R package Gitter.^53^ Using a similar procedure, trait variation within the “sake × tecc” segregating population was estimated on 12 conditions using 3 biological replicates (Tables S5).

### High-throughput CLS assay

High-throughput CLS assays were performed as described previously.^6^ SIs, corresponding to the quantifiable parameter of CLS, were calculated using the CATHODE software.^6^ For each strain or condition tested, three biological replicates were used.

### Linkage mapping

Details for both ISA and BSA methodologies can be found in Supplementary Information.

### Candidate gene validation

Functional validations of candidate genes were performed using either non-complementation or allele replacement approaches.^34^ For the non-complementation strategy, each parental strain of the “sake × tecc cross” was crossed with the BY4741 strain (or BY4742) or its isogenic counterpart carrying a deletion of the gene of interest. To limit problems related to secondary mutations in the deletion collection, each collection strain (BY4741 genetic background) was backcrossed with the BY4742 strain to obtain haploid strains in both BY4741 and BY4742 backgrounds. The strains used for the allele replacement approach (including appropriate control strains) were kindly provided by Aimée Dudley and obtained by fusing either the KanMX or NatMX cassette to the allele of interest prior to transformation into the parental strains.^27^

### Metabolite analyses

Extracellular acetate concentrations were determined using GC-MS. Intracellular metabolite concentrations were obtained using LC-MS. All experimental details are given in Supplementary Information.

### Statistical analyses

Statistical significance of CLS differences between strains and/or conditions was estimated using *t*-test or Welch’s *t*-test depending on variance comparison (through Fisher’s F-test). Normality of SI distribution was tested using the Shapiro–Wilk test where the null hypothesis corresponds to a normal distribution.

Details about heritability analyses are given in Supplementary Information.

### Data availability

Data are available on demand. All sequencing data have been deposited in the European Nucleotide Archive under the accession number PRJEB22383.

## Electronic supplementary material

Supplementary information

Table S1

Table S8

## Electronic supplementary material

**Supplementary Information** accompanies the paper on the *npj Aging and Mechanisms of Disease* website (10.1038/s41514-018-0022-6).
