# Supplementary material for: Natural variation of chronological aging in the Saccharomyces cerevisiae species reveals diet-dependent mechanisms of life span control
Source: NPJ Aging Mech Dis. 2018 Mar 12;4:3. doi: 10.1038/s41514-018-0022-6 (PMC5845861; doi:10.1038/s41514-018-0022-6)
Supplement: Supplementary file 1 — Supplementary information [file 41514_2018_22_MOESM1_ESM.pdf]

Supplementary Information

Manuscript # NPJAMD-00150R

Natural variation of chronological aging in the *Saccharomyces cerevisiae* species reveals diet-dependent mechanisms of life span control

Running title: Aging variation in a yeast population

Paul P. Jung, Zhi Zhang, Nicole Paczia, Christian Jaeger, Tomasz Ignac, Patrick May, Carole L. Linster[*]

Luxembourg Centre for Systems Biomedicine, University of Luxembourg, Esch-sur-Alzette, Luxembourg

[*]Corresponding author:

Email: carole.linster@uni.lu

Phone: +352 46 66 44 6231

Table of Contents

Supplementary Methods 3
Linkage mapping 3
Heritability analysis 3
Short chain fatty acid extraction, derivatization, and GC-MS measurement 3
Quantification of intracellular amino acids and trehalose by LC-MS 4
References 5

Supplementary Tables 6
Supplementary Table S2 Statistical significance of the pairwise CLS comparison in the natural strains collection for different conditions. 6
Supplementary Table S3 Pearson coefficients calculated for pairwise comparison of chronological life span data obtained for our natural strain collection in different conditions. 7
Supplementary Table S4 Environmental conditions used for trait profile analysis in the natural strain collection. 8
Supplementary Table S5 Environmental conditions used for trait profile analysis in the segregant collection. 9
Supplementary Table S6 pH values measured in the medium of 3 day-old cultures in four conditions (2% Glucose (SC), 0.5% Glucose (CR), 10% Glucose (Glu10) and 2% Galactose (Gal)) for the YO486, YO502, FY4 and FY4 $ser1_{YO486}$ strains. 9
Supplementary Table S7 Composition of the Synthetic Complete (SC) medium used in this study. 10

Supplementary Figures 11
Fig. S1 Growth variation within our natural strain collection. 11
Fig. S2 CLS variation within the 'sake x tecc cross' progeny. 12
Fig. S3 Condition-specific correlation between CLS and growth parameters in the 'sake x tecc cross' progeny. 13
Fig. S4 Transgressive segregation of the CLS trait within the 'sake x tecc cross' progeny. 14
Fig. S5 Testing of *MIL1* as an aging gene by a non-complementation approach. 15
Fig. S6 Effect of the *SER1* alleles of the 'sake x tecc cross' parental strains on growth in different conditions. 16
Fig. S7 Impact of extracellular factors on aging. 17
Fig. S8 Metabolome variation during aging between the FY4 and FY4$ser1_{YO486}$ strains. 18
Fig. S9 Serine is the second most used amino acid in the yeast proteome. 19

Supplementary Methods

Linkage mapping

The R/qtl work package was used to carry out Individual Segregant Analysis, considering that 556 genetic markers have been previously identified through RADseq for the entire 'sake x tecc cross' progeny.[1] The threshold at 5% significance level was determined using 1000 permutations. For Bulk Segregant Analysis, the 50 longest- and the 50 shortest-living strains were selected and grown independently, and then pooled using equal amounts of cells from each strain. Total genomic DNAs of the pools were extracted using QIAGEN Genomic-tip 20/G according to the manufacturer's instructions. Genomic Illumina sequencing libraries were prepared with a mean insert size of 280 bp. In total, eight bulks (2 bulks for each tested condition) were multiplexed and sequenced using the Illumina HiSeq2000 system with single-end 50 bp reads. The total number of reads for each sample is given in Table S8. Genetic markers were defined as the nucleotide positions that differ between the haploid YO486 and YO502 strains with a coverage of at least 10x and called by a custom next generation sequencing analysis pipeline, which includes quality control, preprocessing for reads, and mapping to the standard *S. cerevisiae* reference genome (UCSC release SacCer3; BWA, version: 0.7.12; SAMtools, version 0.1.18).[2,3] In total, 47,770 high-confidence SNPs were identified between the haploid YO486 and YO502 strains. The allele frequencies at each genetic marker in the pools were calculated using the pileup files generated from SAMtools.[3] The statistical and analytical framework based on smoothed G statistics (G') described by Magwene and colleagues[4] was performed with a smoothing default window size W = 33,750 kb. A QTL region was defined as a continuous run of at least 10 SNPs spanning at least a 10 kb interval, where the G' statistic exceeded the false discovery rate threshold 0.05. Peaks were identified as contiguous subregions such that $G_i' = 0.95G_{max}'$, where G_{max}' is the site that has the largest G' within the QTL region.[5] Synonymous, non-synonymous, non-sense and frameshift mutations within each QTL region were annotated functionally using ANNOVAR.[6]

Heritability analysis

Broad-sense heritability H^2 is defined as the phenotypic variance (V_P) explained by the genetic variance (V_G). This latter parameter is partitioned as the sum of additive genetic factors (V_A), dominance effects (V_D), gene-gene interactions (V_I) and gene-environment interactions (V_E).[7,8] In this study, broad-sense heritability of the CLS phenotype was estimated as $H^2 = 1 - \frac{\sigma_p^2}{\sigma_s^2}$, where σ_p^2 is the pooled phenotypic variance of the parental strains and σ_s^2 is the pooled phenotypic variance of the segregants (progeny of the 'sake x tecc cross').[9] By contrast, narrow-sense heritability h^2 only takes into account the effect of additive genetic factors (V_A) on phenotype variance (V_P). The h^2 was estimated using a parent-offspring regression, calculated based on the average results obtained for multiple phenotypes for the parents and for the progeny.[7] The conditions used for phenotyping the progeny and parental strains of the 'sake x tecc cross' on solid agar plates are listed in supplementary Table S5.

Short chain fatty acid extraction, derivatization, and GC-MS measurement

This method was used to quantify acetic acid in media of aging yeast cultivations. The extraction method was based on a protocol for short chain fatty acids from Moreau et al.[10] Briefly, 20 µL of the internal standard (2-Ethylbutyric acid, c = 20 mmol/L) were added to 180 µL of medium. The samples were acidified with 10 µL of 37% hydrochloric acid, incubated for 15 min at 15 °C (Eppendorf Thermomixer). Then, 1 mL of diethyl ether was added and the samples were again vortexed for 15 min at 15 °C. The upper organic phase was separated by centrifugation (5 min, 21,000 x g and 15 °C) and 900 µL were collected in a new reaction tube. Again, 1 mL of diethyl ether

were added to the medium, incubated (5 min) and separated by centrifugation. 900 µL of the organic phase were combined with the first extract. Of this preparation, 250 µl were transferred into a GC glass vial with micro insert in triplicates. For derivatization, 25 µL of N-tert-Butyldimethylsilyl-N-methyltrifluoroacetamide with 1% tert-Butyldimethylchlorosilane (Restek) were added and the samples were incubated for a minimum of 1 hour at room temperature. For absolute quantification, an external calibration curve including all compounds of interest (Volatile Free Acid Mix, CRM46975, Sigma-Aldrich) was prepared, extracted, and derivatized as described before.
GC-MS analysis was performed by using an Agilent 7890A GC coupled to an Agilent 5975C inert XL Mass Selective Detector (Agilent Technologies). A sample volume of 1 µl was injected into a Split/Splitless inlet, operating in split mode (20:1) at 270 °C. The gas chromatograph was equipped with a 30 m (I.D. 250 µm, film 0.25 µm) DB-5MS capillary column (Agilent J&W GC Column). Helium was used as carrier gas with a constant flow rate of 1.4 ml/min. The GC oven temperature was held at 80 °C for 1 min and increased to 150 °C at 10 °C/min. Then, the temperature was increased at 50 °C/min to 280 °C and held for 1.4 min. The total run time was 15 min. The transfer line temperature was set to 280 °C. The mass selective detector (MSD) was operating under electron ionization at 70 eV. The MS source was held at 230 °C and the quadrupole at 150 °C. The detector was switched off during elution of MTBSTFA. For quantification, measurements of the compounds of interest were performed in selected ion monitoring mode.
All GC-MS chromatograms were processed using MetaboliteDetector, v3.020151231Ra.[11] The software package supports automatic deconvolution of all mass spectra. Compounds were annotated by retention time and mass spectrum. The data set was normalized by using the response ratio of the integrated peak area of the analyte and the integrated peak area of the internal standard.

Quantification of intracellular amino acids and trehalose by LC-MS
0.5 ml aliquots were taken from the aging yeast cultivations and quenched by the addition of 1.5 ml of 60 % (v/v) methanol at -60 °C. After mixing, cells were separated by centrifugation (10 min, 10,000 x g, -10 °C). The supernatants were carefully removed using a syringe equipped with a needle and the cell pellets stored at -80 °C. In parallel, the cell number and biovolume of each sampled culture was determined using a Coulter Counter (Multisizer Z3, Beckmann Coulter) to calculate the biovolume of the cells in the pellet.
Intracellular metabolites were extracted from the frozen cell pellets by addition of a 200-fold volume of extraction fluid (50 % methanol, 50 % TE buffer at pH 7.0 containing 10 mM TRIZMA and 1 mM EDTA) at -20 °C and an equal volume of chloroform at -20 °C. The mixture was mixed at -20 °C in an Eppendorf shaker for 2 h and then centrifuged for 10 min at 10,000 x g and -10 °C. The upper aqueous phase was taken up, filtered (0.22 µm, PTFE membrane) and stored at -20 °C until LC-MS analysis.
Intracellular metabolites were analyzed by scheduled multi reaction monitoring LC-MS/MS on a Nexera XR LC 20AD (Shimadzu) coupled to an API4000 QTrap mass spectrometer equipped with an electrospray ion source (Sciex). Nitrogen was supplied by an NMG33 generator (CMC). Absolute concentrations were calculated based on external calibration curves prepared in 50 % LC-MS-grade methanol. Measurement values were normalized to ^{13}C-labeled internal standards. Trehalose and intracellular amino acids (serine, glycine and cysteine) were separated by hydrophilic interaction chromatography using a zwitter ionic column (SeQuant®ZIC®-HILIC, Merck) equipped with a pre-column (SecurityGuard ULTRA UHPLC HILIC, Phenomenex). For the analysis of trehalose, an isocratic method at a constant flow rate of 0.7 ml/min, an oven temperature of 55 °C, and a mobile phase consisting in 85 % acetonitrile in water was used. Amino acids were separated at a constant flow rate of 0.25 ml/min, an oven temperature of 25 °C, and using the following gradient where solvent A was water:acetonitrile:formic acid (98.9:1:0.1) and solvent B was acetonitrile:water:formic acid (98.9:1:0.1): 0 min – 90 % B, 2 min - 90 % B, 7 min – 20 % B, 10 min – 10 % B, 12 min – 10 % B, 12.1 min – 90 % B, 20 min – 90 % B. The injection volume was 5 µl of sample for trehalose and 1 µl of sample for amino acids. Mass spectral data were obtained in positive ionization scheduled multi reaction monitoring mode for all analytes.

References

1 Sirr, A. *et al.* Natural Variation in SER1 and ENA6 Underlie Condition-Specific Growth Defects in Saccharomyces cerevisiae. *G3 (Bethesda)*, doi:10.1534/g3.117.300392 (2017).

2 Li, H. & Durbin, R. Fast and accurate short read alignment with Burrows-Wheeler transform. *Bioinformatics* **25**, 1754-1760, doi:10.1093/bioinformatics/btp324 (2009).

3 Li, H. *et al.* The Sequence Alignment/Map format and SAMtools. *Bioinformatics* **25**, 2078-2079, doi:10.1093/bioinformatics/btp352 (2009).

4 Magwene, P. M., Willis, J. H. & Kelly, J. K. The statistics of bulk segregant analysis using next generation sequencing. *PLoS Comput Biol* **7**, e1002255, doi:10.1371/journal.pcbi.1002255 (2011).

5 Granek, J. A., Murray, D., Kayrkci, O. & Magwene, P. M. The genetic architecture of biofilm formation in a clinical isolate of Saccharomyces cerevisiae. *Genetics* **193**, 587-600, doi:10.1534/genetics.112.142067 (2013).

6 Wang, K., Li, M. & Hakonarson, H. ANNOVAR: functional annotation of genetic variants from high-throughput sequencing data. *Nucleic Acids Res* **38**, e164, doi:10.1093/nar/gkq603 (2010).

7 Visscher, P. M., Hill, W. G. & Wray, N. R. Heritability in the genomics era--concepts and misconceptions. *Nat Rev Genet* **9**, 255-266, doi:10.1038/nrg2322 (2008).

8 Bloom, J. S., Ehrenreich, I. M., Loo, W. T., Lite, T. L. & Kruglyak, L. Finding the sources of missing heritability in a yeast cross. *Nature* **494**, 234-237, doi:10.1038/nature11867 (2013).

9 Brem, R. B., Yvert, G., Clinton, R. & Kruglyak, L. Genetic dissection of transcriptional regulation in budding yeast. *Science* **296**, 752-755, doi:10.1126/science.1069516 (2002).

10 Moreau, N. M. *et al.* Simultaneous measurement of plasma concentrations and 13C-enrichment of short-chain fatty acids, lactic acid and ketone bodies by gas chromatography coupled to mass spectrometry. *J Chromatogr B Analyt Technol Biomed Life Sci* **784**, 395-403 (2003).

11 Hiller, K. *et al.* MetaboliteDetector: comprehensive analysis tool for targeted and nontargeted GC/MS based metabolome analysis. *Anal Chem* **81**, 3429-3439, doi:10.1021/ac802689c (2009).

Supplementary Tables

Supplementary Table S2 Statistical significance of the pairwise CLS comparison in the natural strains collection for different conditions.

The p-values shown were determined by a two sample t-test in case of equal variances or a Welch's two-sample t-test in case of unequal variances (indicated by an asterisk).

**CR**							
**SC**	$2.2x10^{-16}$*						
**Glu10**	$2.2x10^{-16}$*	$1.5x10^{-6}$*					
**YNB**	$2.2x10^{-16}$*	0.94	$3.78x10^{-6}$				
**Galactose**	$2.2x10^{-16}$*	0.69	0.0001	0.75			
**Maltose**	0.0048	0.0001	$1.58x10^{-8}$	0.0002	0.0001		
**Raffinose**	0.42*	$2.2x10^{-16}$	$2.2x10^{-16}$	$2.2x10^{-16}$	$2.2x10^{-16}$	0.12	
**Conditions**	**CR**	**SC**	**Glu10**	**YNB**	**Galactose**	**Maltose**	**Raffinose**

CR, calorie restriction (0.5% glucose in SC medium); SC, 2% glucose in SC medium; Glu10, 10% glucose in SC medium; Galactose, 2% galactose in SC medium; Maltose, 2% maltose in SC medium; Raffinose, 2% raffinose in SC medium; YNB, 2% glucose in YNB medium.

Supplementary Table S3 Pearson coefficients calculated for pairwise comparison of chronological life span data obtained for our natural strain collection in different conditions.

Numbers found in parentheses are the p-values corresponding to the regression line.

**CR**							
**SC**	-0.059 (0.6642)						
**Glu10**	0.11 (0.4099)	0.43 (0.0008)					
**YNB**	0.32 (0.088)	0.20 (0.1378)	-0.18 (0.1754)				
**Galactose**	0.25 (0.063)	0.48 (0.00015)	-0.006 (0.9656)	0.44 (0.0007)			
**Maltose**	-0.19 (0.1514)	0.26 0.0048)	0.15 (0.2583)	-0.08 (0.538)	0.07 (0.6235)		
**Raffinose**	0.52 ($3.34\ 10^{-5}$)	0.16 (0.23)	0.13 (0.3167)	0.32 (0.017)	0.38 (0.0037)	-0.03 (0.8447)	
**Conditions**	**CR**	**SC**	**Glu10**	**YNB**	**Galactose**	**Maltose**	**Raffinose**

CR, calorie restriction (0.5% glucose in SC medium); SC, 2% glucose in SC medium; Glu10, 10% glucose in SC medium; Galactose, 2% galactose in SC medium; Maltose, 2% maltose in SC medium; Raffinose, 2% raffinose in SC medium; YNB, 2% glucose in YNB medium.

Supplementary Table S4 Environmental conditions used for trait profile analysis in the natural strain collection.

If not otherwise indicated, growth assays were carried out on solid YPD medium with 2% glucose, supplemented or not with various compounds at the indicated concentrations. The classification "Carbon utilization" indicates that glucose was added at different concentration or substituted by alternative carbon sources at the indicated concentrations.

Supplement	Features	Class
**Glucose**	0.5% - 2% (YPD) - 5% - 10%	Carbon utilization
**Galactose**	2%	Carbon utilization
**Raffinose**	2%	Carbon utilization
**Glycerol**	3%	Carbon utilization
**Ethanol**	3%	Carbon utilization
**Maltose**	2%	Carbon utilization
**Heat shock at 60°C**	10 – 30 – 60 minutes	Environment & Metabolites
**Heat**	37°C	Environment & Metabolites
**NaCl**	1M	Environment & Metabolites
**Ethanol**	10 – 15%	Environment & Metabolites
**Caffeine**	2 – 3mg/ml	Environment & Metabolites
**DMSO**	6%	Toxins
**SDS**	0.003 – 0.005 – 0.01%	Toxins
**H_2O_2**	1 – 5 – 10 – 25mM	Toxins

Supplementary Table S5 Environmental conditions used for trait profile analysis in the segregant collection.

If not otherwise indicated, growth assays were carried out on solid YPD medium with 2% glucose, supplemented or not with various compounds at the indicated concentrations. The classification "Carbon utilization" indicates that glucose was added at different concentration or substituted by alternative carbon sources at the indicated concentrations.

Supplement	Features	Class
**Glucose**	0.5% - 2% (YPD) - 10%	Carbon utilization
**Galactose**	2%	Carbon utilization
**Glycerol**	3%	Carbon utilization
**Ethanol**	3%	Carbon utilization
**Maltose**	2%	Carbon utilization
**NaCl**	1M	Environment & Metabolites
**Ethanol**	10 %	Environment & Metabolites
**DMSO**	8%	Toxins
**H_2O_2**	5 – 10mM	Toxins

Supplementary Table S6 pH values measured in the medium of 3 day-old cultures in four conditions (2% Glucose (SC), 0.5% Glucose (CR), 10% Glucose (Glu10) and 2% Galactose (Gal)) for the YO486, YO502, FY4 and FY4 $ser1_{YO486}$ strains.

The results shown are means ± SDs of three biological replicates. Initial pH values correspond to the pH of the media before inoculation with yeast cells.

Strain	2% Glucose (SC)	0.5% Glucose (CR)	10% Glucose (Glu10)	2% Galactose (Gal)
**Initial pH**	4.14	4.12	4.20	4.11
**YO486**	3.17 (+/- 0.095)	4.51 (+/- 0.24)	2.47 (+/- 0.01)	2.60 (+/- 0.09)
**YO502**	2.85 (+/- 0.06)	4.10 (+/- 0.05)	2.39 (+/- 0.02)	2.66 (+/- 0.025)
**FY4**	3.12 (+/- 0.21)	3.59 (+/- 0.17)	2.53 (+/- 0.005)	3.11 (+/- 0.24)
**FY4$ser1_{YO486}$**	3.05 (+/- 0.025)	3.86 (+/- 0.45)	2.7 (+/- 0.008)	3.05 (+/- 0.03)

Supplementary Table S7 Composition of the Synthetic Complete (SC) medium used in this study.

The listed compounds were added at the indicated final concentrations to minimal YNB medium containing ammonium sulfate as nitrogen source and different sugars (glucose, galactose, maltose or raffinose) as carbon source.

Compound	Concentration (g/l)
**Adenine**	0.04
**Arginine**	0.17
**Aspartate**	0.17
**Glutamate**	0.17
**Histidine**	0.17
**Leucine**	0.35
**Lysine**	0.17
**Methionine**	0.17
**Phenylalanine**	0.17
**Serine**	0.17
**Threonine**	0.17
**Tryptophan**	0.17
**Tyrosine**	0.17
**Valine**	0.17
**Uracil**	0.17
**Alanine**	0.17
**Asparagine**	0.17
**Cysteine**	0.17
**Glutamine**	0.17
**Myo-inositol**	0.17
**Isoleucine**	0.17
**Aminobenzoic acid**	0.017
**Proline**	0.17

Supplementary Figures

Fig. S1

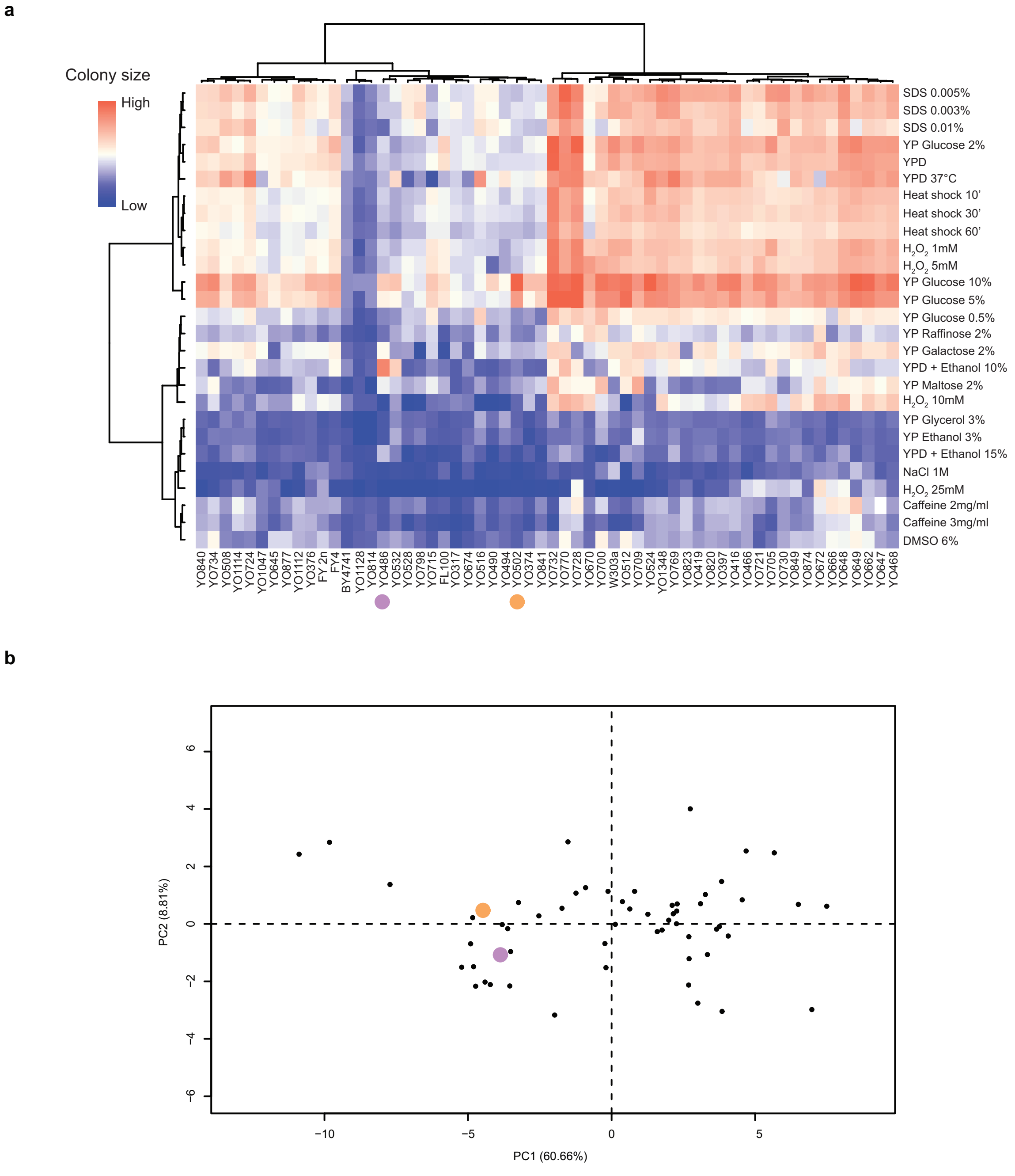

Fig. S1 Growth variation within our natural strain collection.

Colony growth phenotyping within our natural strain collection was performed in the 26 indicated conditions, followed by hierarchical clustering (using a centered Pearson correlation metric and average linkage mapping) (**a**) and Principal Component Analysis (**b**). YPD and YP + Glucose 2% correspond to independent replicates. The parental strains of the 'sake x tecc cross' are indicated by large dots (purple for YO486 and orange for YO502).

Fig. S2

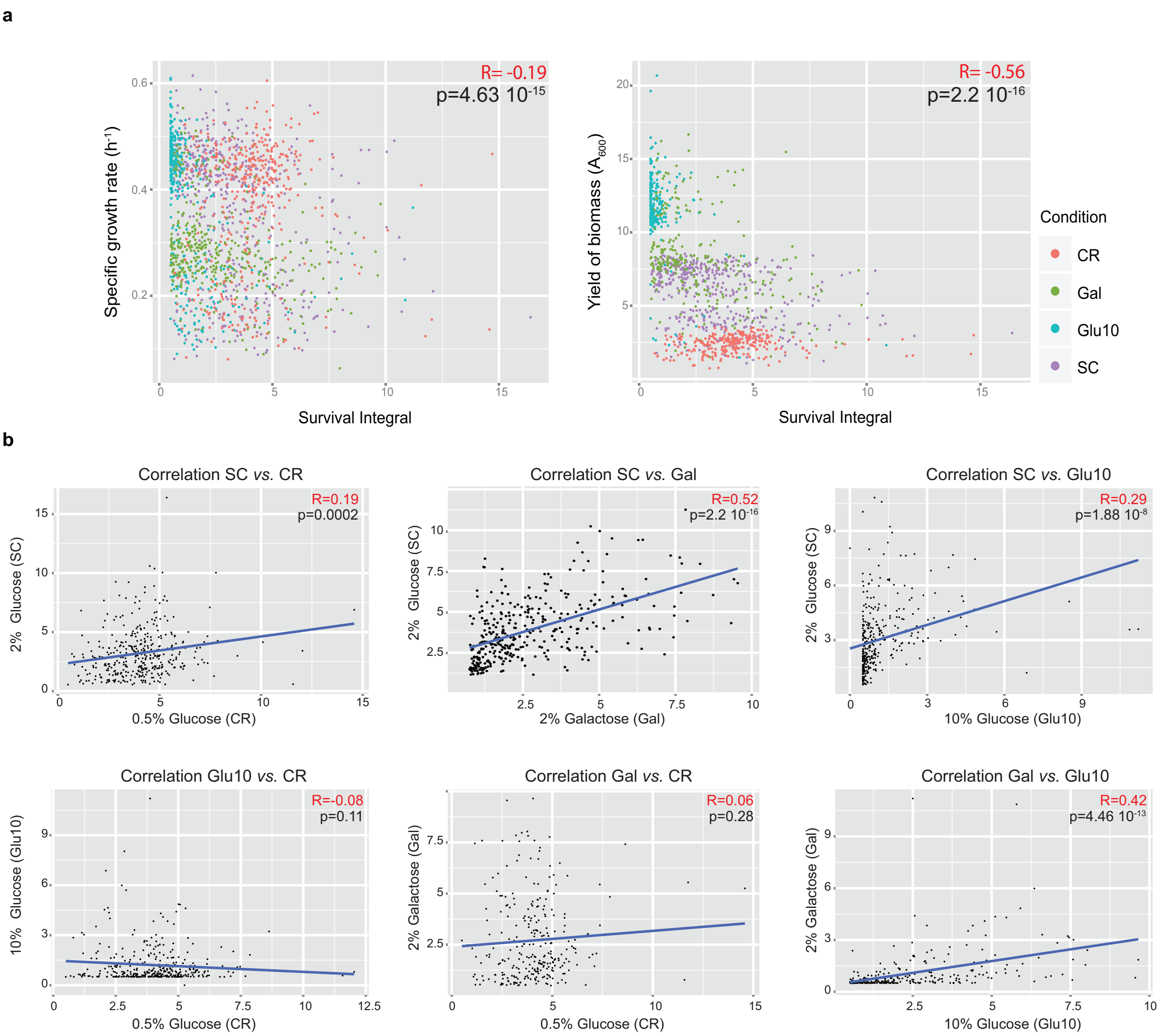

Fig. S2 CLS variation within the 'sake x tecc cross' progeny.

The entire segregating population was subjected to CLS quantification in four environmental conditions. **a** Growth characteristics were also determined for this progeny in the same conditions and CLS was plotted against the specific growth rate (left panel) and the yield of biomass (right panel). The Pearson coefficients (R) and the corresponding p-values (p) were calculated for each plot. **b** Pairwise comparisons of the survival integrals determined in the four conditions and linear regression analysis to calculate the Pearson coefficients (R) and the corresponding p-values (p).

Fig. S3

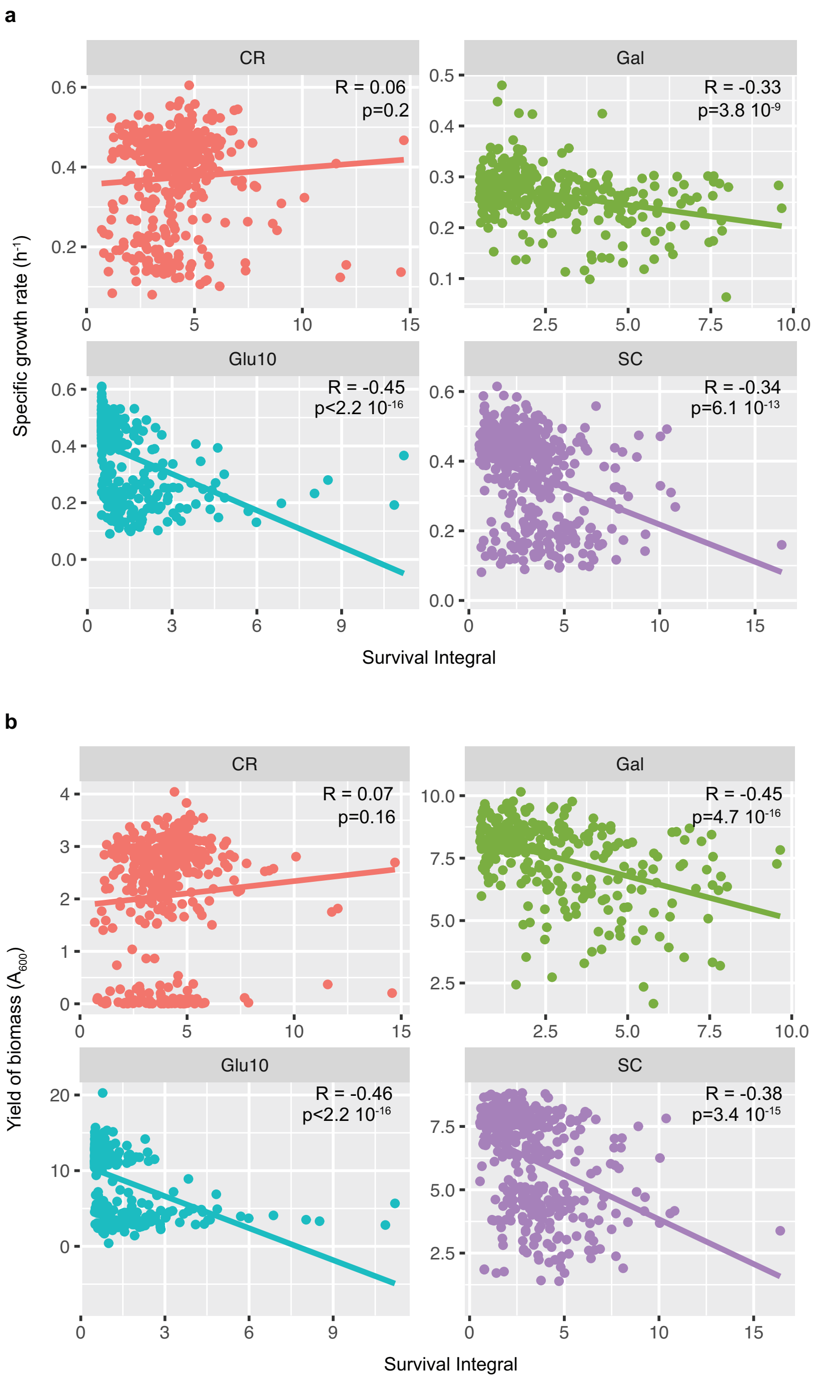

Fig. S3 Condition-specific correlation between CLS and growth parameters in the 'sake x tecc cross' progeny.

Pearson coefficients (R) and the corresponding p-values (p) were calculated between CLS and specific growth rate (**a**) and yield of biomass (**b**) for each condition individually.

Fig. S4

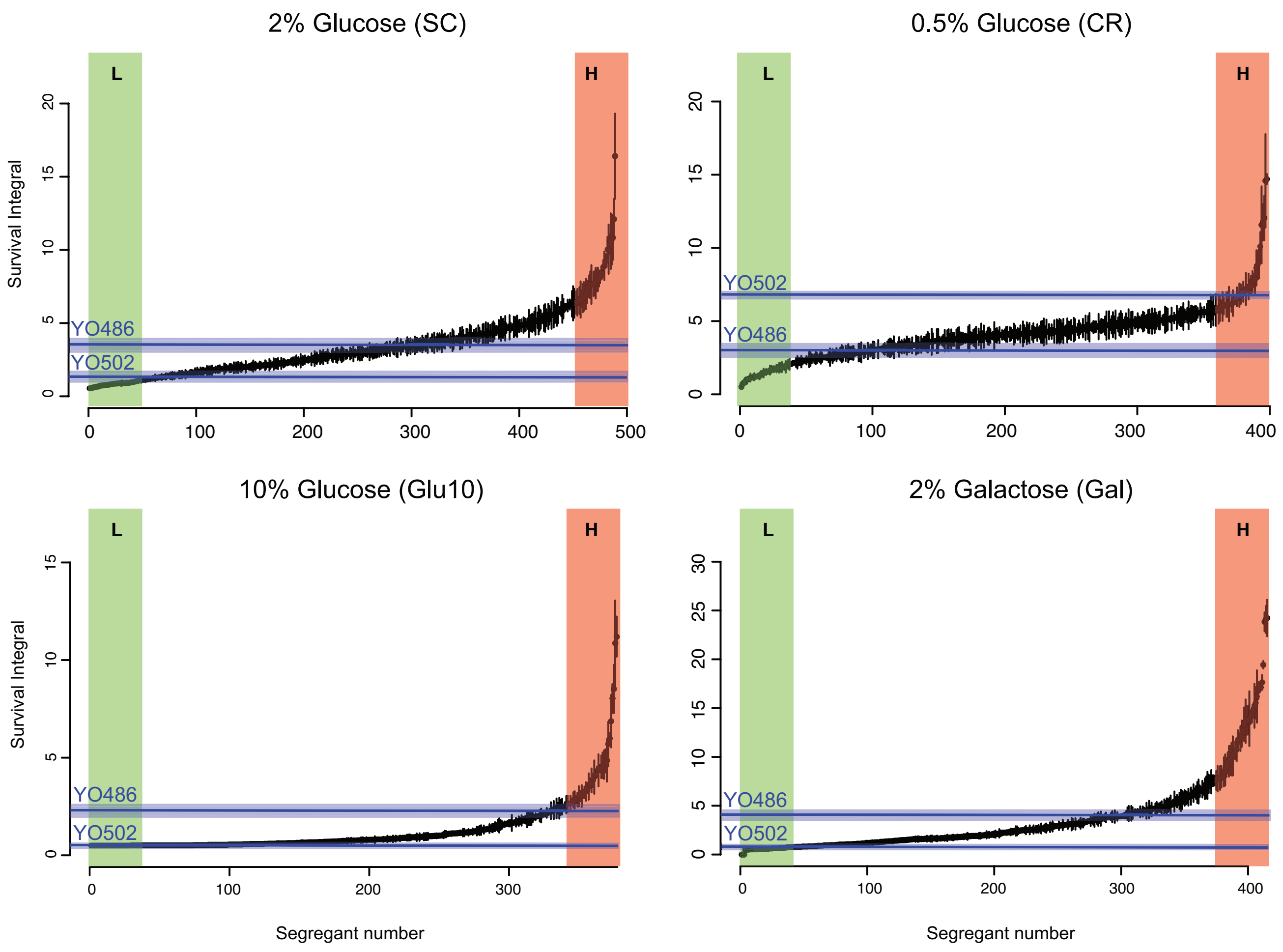

Fig. S4 Transgressive segregation of the CLS trait within the 'sake x tecc cross' progeny.

Ascending representation of survival integrals determined for the entire progeny in the four indicated conditions. Blue lines correspond to the survival integral values (mean of 3 biological replicates) obtained for the YO486 and YO502 strains, the surrounding lighter blue areas representing the standard deviations. Green (L) and red (H) rectangles indicate the short- and long-lived strain bulks used for BSA analyses.

Fig. S5

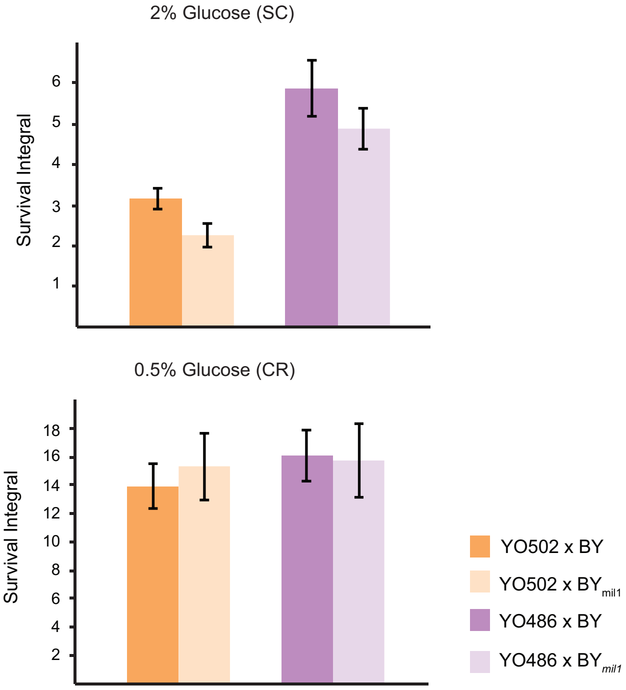

Fig. S5 Testing of *MIL1* as an aging gene by a non-complementation approach.

Survival integrals were determined using an outgrowth kinetics assay for the indicated hybrid strains in SC medium containing 2% glucose and 0.5% glucose (calorie restriction). The results correspond to means ± SDs for 3 biological replicates.

Fig. S6

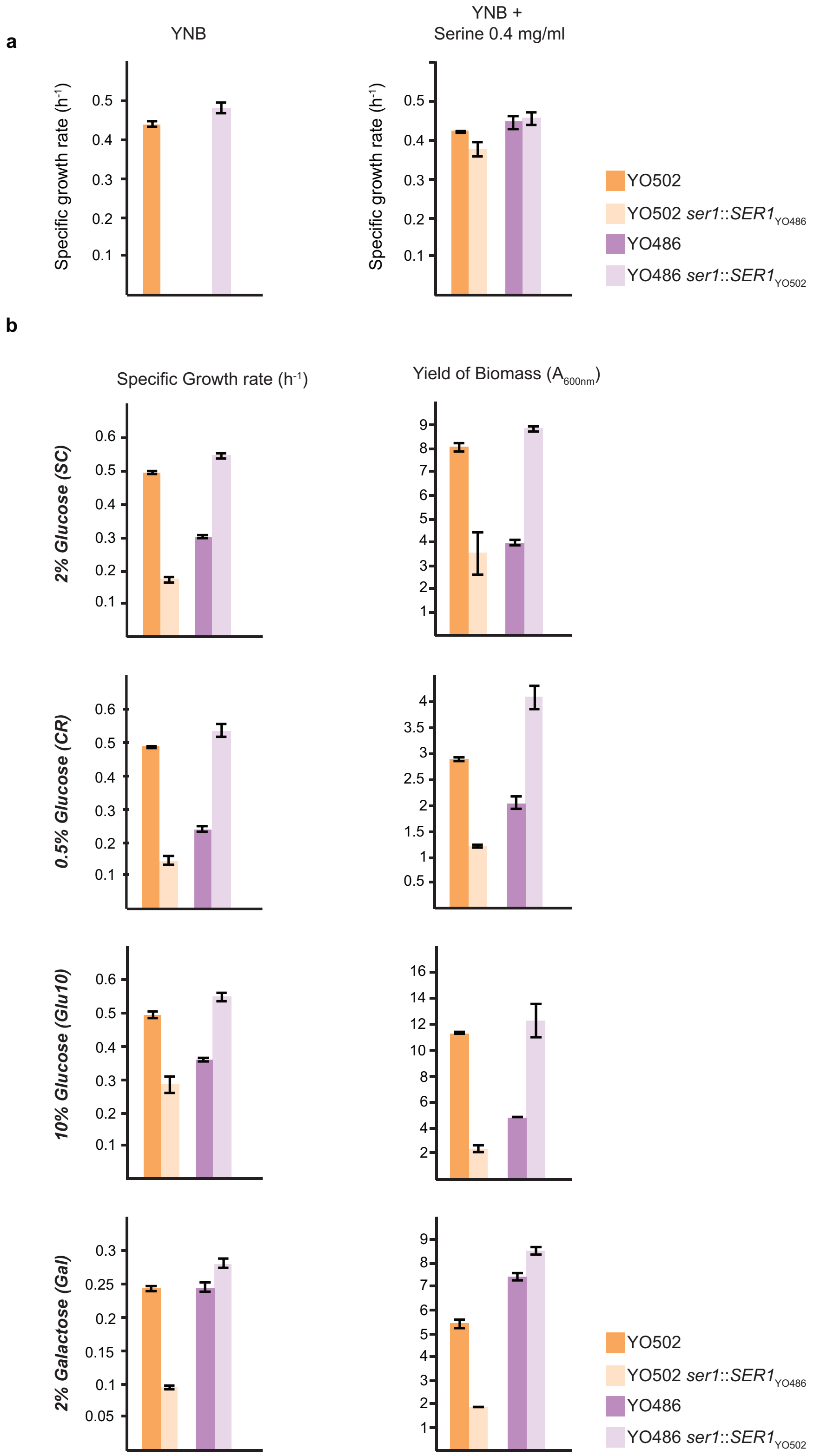

Fig. S6 Effect of the *SER1* alleles of the ‘sake x tecc cross’ parental strains on growth in different conditions.

SER1 allele-swapped strains were compared to the original strains. **a** The effect of serine supplementation on growth was tested in minimal YNB medium containing 2% glucose. **b** Growth rate and yield of biomass were also investigated in the four conditions used for the CLS studies. In all of these conditions, both growth parameters differed significantly between control and allele-swapped

strains (p-value < 0.05 according to Student's t-test). All results shown correspond to means ± SDs for 3 biological replicates.

Fig. S7

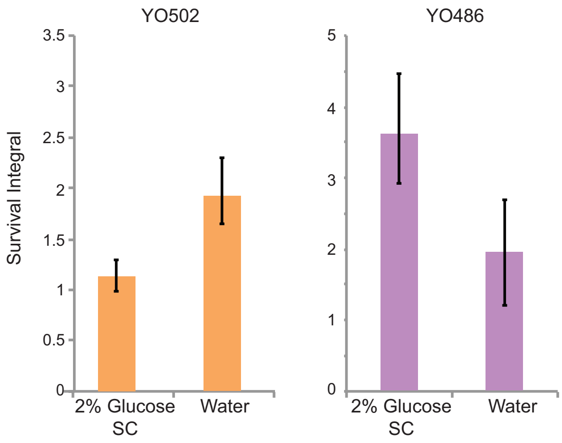

Fig. S7 Impact of extracellular factors on aging.

CLS was measured in YO502 (orange) and YO486 (violet) cultivations started in SC (2% glucose) medium and switched (or not) on day 3 to water. All results shown correspond to means ± SDs for 3 biological replicates.

Fig. S8

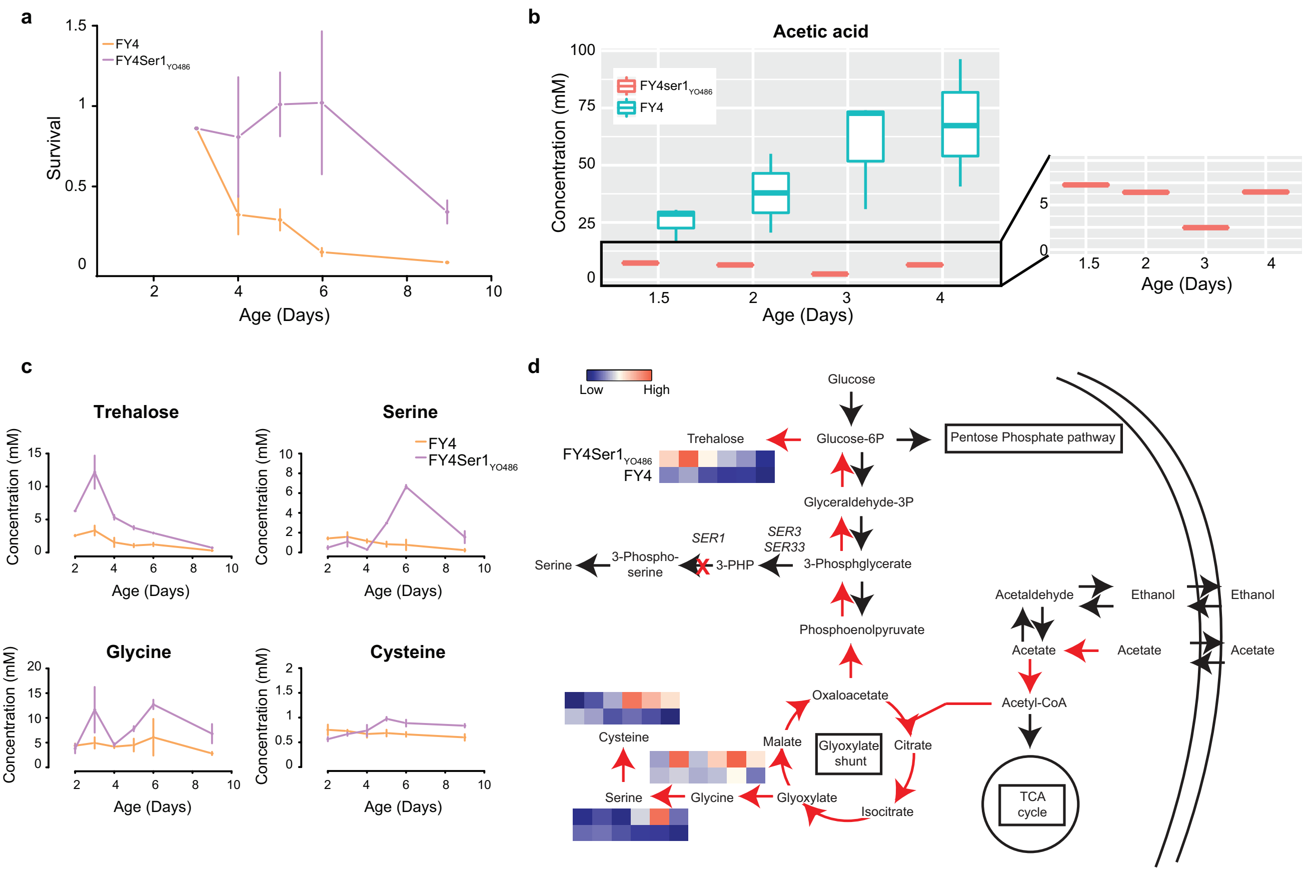

Fig. S8 Metabolome variation during aging between the FY4 and FY4ser1$_{YO486}$ strains.

a CLS profiles obtained for FY4 (orange) and FY4ser1$_{YO486}$ (violet) flask cultivations in SC medium supplemented with 2% glucose.. The aging cultivations were sampled at different time points for endo- and exo-metabolome analyses. **b** Acetic acid was quantified by GC-MS in the extracellular medium of the FY4 (in blue) and FY4ser1$_{YO486}$ (in red) strains. **c** Intracellular trehalose, serine, glycine, and cysteine concentrations were also determined during aging in the two strains using LC-MS. **d** Schematic overview showing the metabolites quantified in this study within the known metabolic network of *S. cerevisiae*. The endo-metabolome data is represented by heatmaps where red and blue squares correspond to high and low amounts of each metabolite, respectively, at the different time points. Red arrows highlight reactions that may be more active in the FY4ser1$_{YO486}$ strain due to a metabolic reprogramming in response to *SER1* deficiency and that could explain lower acetate accumulation, higher trehalose levels and increased CLS in this strain. The main serine synthesis pathway, starting from 3-phosphoglycerate, is also shown. The first and second reactions in this pathway are catalysed by the Ser3 (or its paralog Ser33) and Ser1 enzymes, respectively. It is the latter enzyme that is deficient in the FY4ser1$_{YO486}$ strain (as indicated by the red cross). 3-PHP: 3-phosphohydroxypyruvate.

Fig. S9

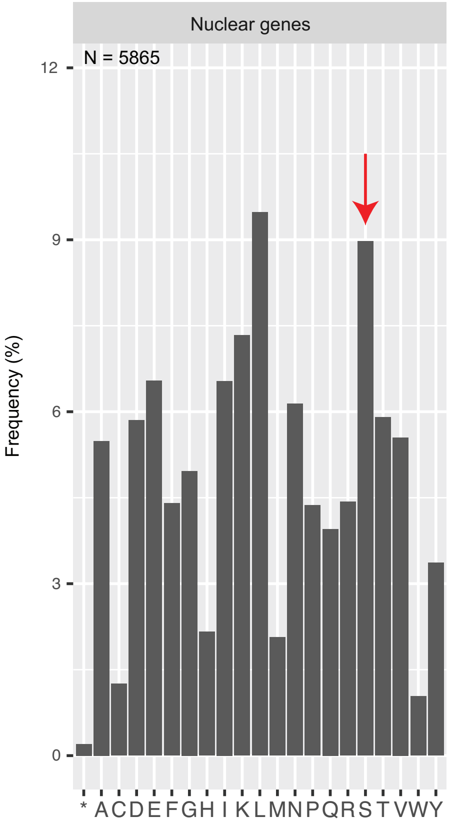

Fig. S9 Serine is the second most used amino acid in the yeast proteome.

In total, 5865 nuclear genes were analysed for codon usage. Data were obtained from http://wiki.yeastgenome.org/index.php/S._cerevisiae_Codon_Usage_Tables. The red arrow indicates serine. All amino acids are represented with the 1-Letter code. * designates a stop codon.
